# Flow diversion for cerebral aneurysms

**DOI:** 10.3171/2022.7.FOCVID2276

**Published:** 2022-10-01

**Authors:** Peter T. Kan, Elad I. Levy, Felipe C. Albuquerque, Mandy Jo Binning

**Affiliations:** 1 Department of Neurosurgery, University of Texas Medical Branch, Houston, Texas;; 2 Departments of Neurosurgery and Radiology, University of Buffalo, New York;; 3 Department of Neurosurgery, Barrow Neurological Institute, Phoenix, Arizona; and; 4 Neurosurgery, Global Neurosciences Institute, Lawrenceville, New Jersey

In the past decade, similar to the operative microscope for aneurysm clipping, Guglielmi detachable coils, and self-expanding intracranial stents for aneurysm coil embolization, flow diversion represents a major advancement in the treatment of cerebral aneurysms and has changed the paradigm from intrasaccular aneurysm treatment with coil embolization to parent vessel reconstruction with flow diversion. Flow diverters are flexible stents with a much higher metal coverage and lower porosity than traditional intracranial stents for aneurysm treatment. They work by redirecting blood away from the aneurysm through the parent artery, leading to aneurysm stasis, progressive thrombosis, and subsequent stent endothelialization, the mechanism for excluding the aneurysm from circulation. Remarkably, flow diverters still allow blood flow through perforators given the demand through these small critical vessels. Since the approval of flow diversion for the treatment of large and giant previously difficult-to-treat carotid sidewall aneurysms,^1^ its indication has expanded to include smaller aneurysms^2^ and other off-label uses, such as posterior circulation aneurysms, distal aneurysms, bifurcation aneurysms, blister aneurysms, and other ruptured aneurysms, with good results. However, despite a decade of experience, giant, partially thrombosed, fusiform vertebrobasilar aneurysms remain a challenging entity for flow diversion in which the results remain relatively poor in comparison,^3^ although newer devices and a better understanding of antiplatelet and anticoagulation management may improve the outcome in this devastating disease. Lastly, although the risk of ischemic strokes associated with flow diversion is now less than 5% for unruptured aneurysms,^2,4,5^ thromboembolic events remain the most common complication. At the same time, dual antiplatelet therapy, a requirement for flow diverter placement, can be associated with bleeding complications and is suboptimal in the setting of subarachnoid hemorrhage. Newer devices with surface modification are emerging to address the unmet need of reducing thrombogenicity and antiplatelet requirements for flow diverters. This issue of *Neurosurgical Focus: Video* includes representative videos that illustrate the spectrum of cases discussed above.

## Disclosures

Dr. Levy: personal fees from NeXtGen Biologics, RAPUID Medical, Claret Medical, Cognition Medical, Imperative Care, Rebound Therapeutics, StimMed, Three Rivers Medical, Medtronic, Penumbra, MicroVention, Integra, Clarion, GLG Consulting, Guidepoint Global, Misionix, Mosiac, Haniva Technology, Endostream, MEDX, and IRRAS AB outside the submitted work; patent pending for Bone Scalpel; leadership or fiduciary role in CNS, ABNS, and UBNS; and rendering of medical/legal opinions as an expert witness.
